# Supplementary material for: Gender and societies: a grassroots approach to women in science
Source: R Soc Open Sci. 2019 Sep 4;6(9):190633. doi: 10.1098/rsos.190633 (PMC6774970; doi:10.1098/rsos.190633)
Supplement: Supplementary Material main document;Supplementary Material Table S1 (TableS1.xlsx) [file rsos190633supp1.docx]

Supplementary Material for: Gender and societies: a grassroots approach to women in science

Contains: Table S1 (TableS1.xlsx) – society data.

Tables S2 – S5 Benchmarking data by country

Figure S1 – Benchmarking data by country, subject and career stage

Full data sources – list of references and URLs from which benchmarking data were collected

**Table S1.** (See TableS1.xlsx) Data from 31 scientific societies spanning four countries and five subject areas. Each row shows data for a single society with columns showing:

- Society: name of society
- Country
- Editors: number of men and women in the associate editor role
- Chief editors: number of men and women in the chief editor role
- Journals: names of publications for which editorial role data were collected
- Late career awardees: number of men and women who have received a late career award since 2000
- Late career awards: names of the awards for which data were collected
- Early career awardees: number of men and women who have received an early career award since 2000
- Early career awards: names of the awards for which data were collected
- Presidents: number of men and women who have been society president since 2000
- Student awardees: number of men and women who have received a student award since 2000
- Student awards: names of the awards for which data were collected

| **United Kingdom** | | | | |
| --- | --- | --- | --- | --- |
|  | **Academic staff** | **Senior staff** | **Junior staff** | **Postgraduate** |
| **Astronomy** | 18% | 11% | 21% | 37% |
|  | Phys academics | Phys P | Phys postdoc | Phys PG |
|  | Institute of Physics^11^ | Institute of Physics^11^ | Institute of Physics^11^ | HESA^9^ |
|  | 2016 | 2016 | 2016 | 2016/17 |
| **Ecology** | 45.7% | 15% | 50.0% | 60% |
|  | Biol academics | Biol P | Biol non-P | Biol PG |
|  | HESA^7^ | HESA^8^ | HESA^7^ | HESA^9^ |
|  | 2017 | 2012 | 2017 | 2016/17 |
| **Economics** | 26.5% | 21.7% | 35.4% | 38% |
|  | Econ L/AP/P | Econ AP/P | Econ postdoc/L/SL | Econ PhD students |
|  | Royal Economic Society^17^ | Royal Economic Society^17^ | Royal Economic Society^17^ | Royal Economic Society^13^ |
|  | 2016 | 2016 | 2016 | 2014 |
| **Mathematics & statistics** | 20.0% | 8.5% | 23.3% | 30% |
|  | Maths L/SL/P | Maths P | Maths postdoc/L/SL | Maths/stats PG |
|  | LMS^12^ | LMS^12^ | LMS^12^ | HESA^9^ |
|  | 2015 | 2015 | 2015 | 2016 |

**Table S2.** Benchmarking data for the UK. For each subject and career level, the table shows the percentage of women in the category given, the data source and the year the data was collected. Abbreviations: PG = postgraduate research student, L = lecturer, SL = senior lecturer, AP = associate professor or reader, P = professor. Superscript numbers are references to full data sources listed below.

| **United States of America** | | | | |
| --- | --- | --- | --- | --- |
|  | **Academic staff** | **Senior staff** | **Junior staff** | **Postgraduate** |
| **Astronomy** | 18% | 15% | 22% | 20% |
|  | Phys/astron academics | Phys/astron AP/P | Phys/astron asstP/other | Phys/astron PhD |
|  | AIP^1^ | AIP^1^ | AIP^1^ | NSF^14^ |
|  | 2010 | 2010 | 2010 | 2016 |
| **Ecology** | 24.5% | 23.5% | 37.9% | 53% |
|  | Biol faculty | Biol AP/P | Biol postdoc/asstP | Biol/biomed PhD |
|  | Sheltzer & Smith^16^ | Sheltzer & Smith^16^ | Sheltzer & Smith^16^ | NSF^14^ |
|  | 2014 | 2014 | 2014 | 2016 |
| **Economics** | 23.5% | 15% | 31% | 34% |
|  | Econ faculty | Econ P | Econ assistant faculty | Econ PhD |
|  | Bayer & Rouse^5^ | Bayer & Rouse^5^ | Bayer & Rouse^5^ | NSF^14^ |
|  | 2016 | 2016 | 2016 | 2016 |
| **Mathematics** | 25.3% | 22.4% | 31.7% | 27% |
|  | Maths tenured/TT | Maths tenured | Maths postdoc/TT | Maths PhD |
|  | AMS^2^ | AMS^2^ | AMS^2^ | NSF^14^ |
|  | 2015 | 2015 | 2015 | 2016 |
| **Statistics** | 23.6% | 19.8% | 29.8% | 40% |
|  | Stats tenured/TT | Stats tenured | Stats postdoc/TT | Stats PhD |
|  | AMS^2^ | AMS^2^ | AMS^2^ | NSF^14^ |
|  | 2015 | 2015 | 2015 | 2016 |

**Table S3.** Benchmarking data for the USA. For each subject and career level, the table shows the percentage of women in the category given, the data source and the year the data was collected. Abbreviations: asstP = assistant professor, TT = tenure-track, AP = associate professor, P = professor, other = “other ranks”. Superscript numbers are references to full data sources listed below.

| **Australia** | | | | |
| --- | --- | --- | --- | --- |
|  | **Academic staff** | **Senior staff** | **Junior staff** | **Postgraduate** |
| **Astronomy** | 19% | 11% | 24% | 39.7% |
|  | Astr. cont. staff | Phys AP/P | Phys postdoc/L/SL | Astronomy PhD |
|  | Decadal plan^19^ | ARC^4^/SAGE^15^ | ARC^4^/SAGE^15^ | AAS^20^ |
|  | 2015 | 2014 | 2014 | 2010 |
| **Ecology** | 37% | 25% | 54% | N/A |
|  | Ecol, workforce | Biol AP/P | Biol postdoc/L/SL |  |
|  | AAS^21^ | ARC^4^/SAGE^15^ | ARC^4^/SAGE^15^ |  |
|  | 2015 | 2014 | 2014 |  |
| **Economics** | 21% | 7% | 30% | N/A |
|  | Econ academics | Econ AP/P | Econ assoc. L/L/SL |  |
|  | Hopkins^10^ | Hopkins^10^ | Hopkins^10^ |  |
|  | 2003 | 2003 | 2003 |  |
| **Mathematics & statistics** | 23% | 14% | 28% | 34% |
|  | Maths/stats level A-E | Maths/stats level D/E | Maths/stats level A-C | Maths/stats PhD |
|  | AMSI^9^ | AMSI^9^ | AMSI^9^ | AMSI^9^ |
|  | 2016 | 2016 | 2016 | 2016 |

**Table S4.** Benchmarking data for Australia. For each subject and career level, the table shows the percentage of women in the category given, the data source and the year the data was collected. Abbreviations: L = lecturer, SL = senior lecturer, AP = associate professor, P = professor. Levels A to C are equivalent to assistant lecturer to senior lecturer; levels D and E are equivalent to associate professor and professor. Superscript numbers are references to full data sources listed below.

| **New Zealand** | | | | |
| --- | --- | --- | --- | --- |
|  | **Academic staff** | **Senior staff** | **Junior staff** | **Postgraduate** |
| **Astronomy** | 13% | 7% | 22% | 33% |
|  | Phys L/SL/AP/P | Phys AP/P | Phys L/SL | Phys/astron PhD |
|  | TEC^18^ | TEC^18^ | TEC^18^ | Education Counts^6^ |
|  | 2012 | 2012 | 2012 | 2017 |
| **Ecology** | 24% | 15% | 33% | 54% |
|  | Ecol L/SL/AP/P | Ecol AP/P | Ecol L/SL | Biol PhD |
|  | TEC^18^ | TEC^18^ | TEC^18^ | Education Counts^6^ |
|  | 2012 | 2012 | 2012 | 2017 |
| **Economics** | 23% | 13% | 30% | 50% |
|  | Econ L/SL/AP/P | Econ AP/P | Econ L/SL | Econ PhD |
|  | TEC^18^ | TEC^18^ | TEC^18^ | Education Counts^6^ |
|  | 2012 | 2012 | 2012 | 2017 |
| **Mathematics** | 14% | 5% | 21% | 47% |
|  | Maths L/SL/AP/P | Maths AP/P | Maths L/SL | Maths/stats PhD |
|  | TEC^18^ | TEC^18^ | TEC^18^ | Education Counts^6^ |
|  | 2012 | 2012 | 2012 | 2017 |
| **Statistics** | 25% | 22% | 26% |  |
|  | Stats L/SL/AP/P | Stats AP/P | Stats L/SL |  |
|  | TEC^18^ | TEC^18^ | TEC^18^ |  |
|  | 2012 | 2012 | 2012 |  |

**Table S5.** Benchmarking data for New Zealand. For each subject and career level, the table shows the percentage of women in the category given, the data source and the year the data was collected. Abbreviations: L = lecturer, SL = senior lecturer, AP = associate professor, P = professor. Superscript numbers are references to full data sources listed below.


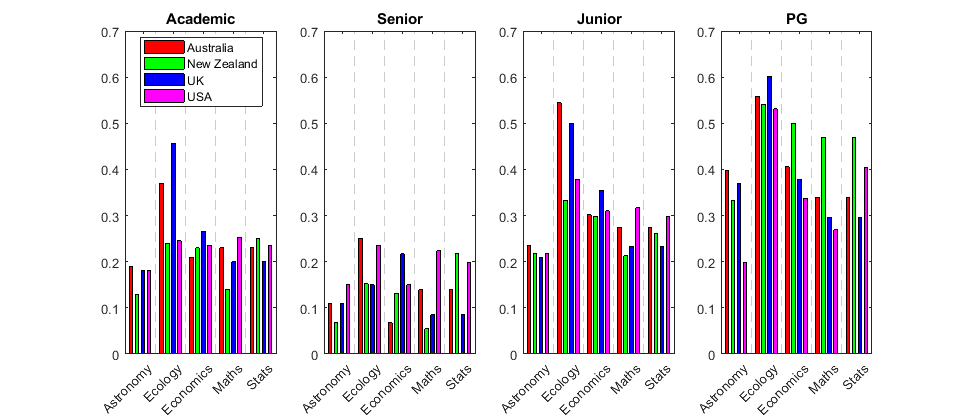


**Figure S1. Benchmarking data for the proportion of women in each subject area by country. Academic** shows all academic staff (senior and junior combined). **Senior** shows senior academic staff, corresponding to Full Professor in the USA, and Associate Professor or Professor in the other countries. **Junior** shows junior academic staff, corresponding to Assistant/Associate Professor in the USA, and Lecturer or Senior Lecturer in the other countries, and includes postdoctoral fellows and research-only staff. **PG** shows postgraduate students. See Tables S2-S5 for details and sources.

**Full data sources** (all URLs accessed on 28 June, 2018)

1. American Institute of Physics: Women among Physics & Astronomy Faculty 2013. <https://www.aip.org/statistics/reports/women-among-physics-astronomy-faculty>
2. American Mathematical Society Conference Board of Mathematical Sciences 2015 Survey Report. <http://www.ams.org/profession/data/cbms-survey/cbms2015>
3. Australian Mathematical Sciences Institute Discipline Profile 2017. <http://amsi.org.au/publications/discipline-profile-mathematical-sciences-2017/>
4. Australian Research Council Gender Snapshot Grants commencing in 2016. <https://www.arc.gov.au/policies-strategies/strategy/gender-snapshot-grants-commencing-2016>
5. Bayer, A. and Rouse, C.E. (2016) Diversity in the Economics Profession: A New Attack on an Old Problem J. Econ. Pers. 30: 221-242. <https://pubs.aeaweb.org/doi/pdfplus/10.1257/jep.30.4.221>
6. Education Counts 2017. <https://www.educationcounts.govt.nz/__data/assets/excel_doc/0015/41703/Provider-based-Equivalent-Full-Time-EFTS-2008-2017-final.xlsx>
7. Higher Education Statistics Agency (HESA) Staff in Higher Education 2016/17. <https://www.hesa.ac.uk/data-and-analysis/staff/cost-centres>
8. Higher Education Statistics Agency (HESA) data in the Royal Society of Biology: women in academic STEM careers 2013. <https://www.rsb.org.uk/images/Society_of_Biology_response_to_women_in_STEM_careers_inquiry.pdf>
9. Higher Education Statistics Agency (HESA): Student Statistics 2016/17. <https://www.hesa.ac.uk/news/11-01-2018/sfr247-higher-education-student-statistics/subjects>
10. Hopkins, S. (2004). Women in economics departments in Australian universities: is there still a gender imbalance? Economic Papers 23, 201-210. <https://onlinelibrary.wiley.com/doi/pdf/10.1111/j.1759-3441.2004.tb00365.x>
11. Institue of Physics acadmic staff in UK Physics departments 2017. <https://www.iop.org/policy/consultations/file_69758.pdf>
12. London Mathematical Society: Women in Mathematics Benchmarking Data 2016. <https://www.lms.ac.uk/sites/lms.ac.uk/files/Benchmarking%20Data%20Updated%20for%202011-2015%20April%202016_0.pdf>
13. Mitka, M., Mumford, K. and Schel, C. (2015). The 10th Royal Economic Society Women’s Committee Survey: The Gender Balance of Academic Economics in the UK 2014. <http://www.res.org.uk/SpringboardWebApp/userfiles/res/file/Womens%20Committee/Biennial%20Report/Gender%20Survey%202014SurveyResults_final.pdf>
14. National Science Foundation Survey of earned doctorates 2016. <https://www.nsf.gov/statistics/2018/nsf18304/data.cfm>
15. Science in Australia Gender Equity (SAGE) 2014. <http://www.sciencegenderequity.org.au/gender-equity-in-stem/>
16. Sheltzer, J.M. and Smith, J.C. (2014) Elite male faculty in the life sciences employ fewer women. Proceedings of the National Academy of Sciences 111: 10107-10112. <http://www.pnas.org/content/pnas/111/28/10107.full.pdf>
17. Tenreyro, S. (2017) Royal Economic Society’s Report on The Gender Balance in UK Economics Departments and Research Institutes in 2016. <http://www.res.org.uk/SpringboardWebApp/userfiles/res/file/Womens%20Committee/Publications/WomensCommitteeReport_2016SurveyResults.pdf>
18. Tertiary Education Commission, Performance-based research fund 2012.
19. Australian Academy of Science. National Committee for Astronomy (2015). *Australia in the era of global astronomy: The decadal plan for Australian astronomy 2016-2025.* Australian Academy of Science.
20. Australian Academy of Science. National Committee for Astronomy (2010). *Mid-term review of the decadal plan for Australian astronomy 2006-2015.* Australian Academy of Science.
21. Australian Academy of Science (2015). *Discovering biodiversity: A decadal plan for taxonomy and biosystematics in Australia and New Zealand 2018-2027.* Australian Academy of Science.
